# LaGAT: link-aware graph attention network for drug–drug interaction prediction

**DOI:** 10.1093/bioinformatics/btac682

**Published:** 2022-10-22

**Authors:** Yue Hong, Pengyu Luo, Shuting Jin, Xiangrong Liu

**Affiliations:** School of Informatics, Xiamen University, Xiamen 361005, China; School of Informatics, Xiamen University, Xiamen 361005, China; School of Informatics, Xiamen University, Xiamen 361005, China; National Institute for Data Science in Health and Medicine, Xiamen University, Xiamen 361005, China; MindRank AI Ltd., Hangzhou 310000, China; School of Informatics, Xiamen University, Xiamen 361005, China; National Institute for Data Science in Health and Medicine, Xiamen University, Xiamen 361005, China

## Abstract

**Motivation:**

Drug–drug interaction (DDI) prediction is a challenging problem in pharmacology and clinical applications. With the increasing availability of large biomedical databases, large-scale biological knowledge graphs containing drug information have been widely used for DDI prediction. However, large knowledge graphs inevitably suffer from data noise problems, which limit the performance and interpretability of models based on the knowledge graph. Recent studies attempt to improve models by introducing inductive bias through an attention mechanism. However, they all only depend on the topology of entity nodes independently to generate fixed attention pathways, without considering the semantic diversity of entity nodes in different drug pair links. This makes it difficult for models to select more meaningful nodes to overcome data quality limitations and make more interpretable predictions.

**Results:**

To address this issue, we propose a Link-aware Graph Attention method for DDI prediction, called LaGAT, which is able to generate different attention pathways for drug entities based on different drug pair links. For a drug pair link, the LaGAT uses the embedding representation of one of the drugs as a query vector to calculate the attention weights, thereby selecting the appropriate topological neighbor nodes to obtain the semantic information of the other drug. We separately conduct experiments on binary and multi-class classification and visualize the attention pathways generated by the model. The results prove that LaGAT can better capture semantic relationships and achieves remarkably superior performance over both the classical and state-of-the-art models on DDI prediction.

**Availabilityand implementation:**

The source code and data are available at https://github.com/Azra3lzz/LaGAT.

**Supplementary information:**

Supplementary data are available at *Bioinformatics* online.

## 1 Introduction

Drug–drug interactions (DDI) mean that when two or more drugs are taken by the patient at the same time, the efficacy of the drugs may be enhanced or weakened, and even side effects may occur. The DDI is a very common situation for patients with complex conditions, it may accelerate the treatment process of the condition, may also affect the health of the patient, and even lead to the death of the patient (Vilar *et al.*, 2014). To reduce medical risks and improve treatment effects, accurate prediction of DDI has become an important clinical task.

Since deep learning is suitable for processing large-scale data and can obtain features with high generalization ability (Ryu *et al.*, 2018), it has been widely used to predict DDI. Some researchers focus on directly exploiting drug-related features such as side effects (Jin *et al.*, 2017; Ma *et al.*, 2018; Zitnik *et al.*, 2018), molecular structure (Asada *et al.*, 2018; Nyamabo *et al.*, 2021; Takeda *et al.*, 2017) and SMILES sequences (Huang *et al.*, 2020) for DDI prediction. These DDI prediction models treat drugs as an independent data sample without considering the topological information of the interactions among drugs. Therefore, many researchers further combine large drug databases with biomedical textual information to construct a biological knowledge graph (KG) to obtain semantic information of drug entities (Gao *et al.*, 2022; Zheng *et al.*, 2021), and to perform DDI prediction combined with drug molecular features.

Existing KG-based models usually use node embedding methods to derive drug node features (Chen *et al.*, 2021), or learn higher-order semantics of nodes through graph neural network (Lin *et al.*, 2020; Yu *et al.*, 2021). Although these methods have contributed for the DDI prediction, they share the common problem that each drug obtains the same embedding representation when making predictions with different drugs. That is, they ignore the semantic diversity of entity nodes in different drug pair links, which limits the model’s ability to further mine knowledge graph data information and make more interpretable predictions.

To address the above limitations, we propose a Link-aware Graph Attention method, named LaGAT, which enables the model to capture different semantic information of drugs from neighborhood nodes in the knowledge graph based on the drug pair link. Specifically, we construct a link-aware graph attention layer as the core of the model. For drug *v* and drug *u* in a drug pair link, the embedded representation of drug *u* is used as a query vector to compute the attention weights of the neighbors of drug *v* to generate different attention paths and update the embedding of each node in the subgraph with the embedding representation of the neighbors. We conduct experiments on two real datasets, binary and multi-class, and selected typical DDI examples to visualize the generated attention pathways of our model. Experimental results show that our model has certain interpretability and outperforms existing state-of-the-art models on both datasets. Our contributions are summarized as follows:

- We propose a novel graph attention method that improves on the limitations of DDI prediction on knowledge graphs. It allows the model to generate different attention pathways for the drug entity by different drug pair links to aggregate neighbor node information.
- Our propose method can make the model interpretable. The LaGAT can make the model pay attention to more meaningful entity nodes, which is beneficial for the model to predict different types of DDIs.
- We evaluate LaGAT on two public datasets and demonstrate its superiority over the state-of-the-art DDI prediction models.

## 2 Related work

*Attention mechanism on graph*. Attention mechanism can be used to improve the prediction performance of neural network models (Bahdanau *et al.*, 2014) and provide a certain degree of interpretability for neural network structures (Vashishth *et al.*, 2019; Wiegreffe and Pinter, 2019), which has become a research hotspot in recent years. There have been some studies trying to promote the attention mechanism on graph data structure. The Graph Attention Network (GAT) (Veličković *et al.*, 2018) incorporates the attention mechanism into the propagation step and the multi-head attention mechanism used by to stabilize the learning process. The Gated Attention Network (GaAN) (Zhang *et al.*, 2018) also uses a multi-head attention mechanism. It uses a gated attention mechanism to collect information from different heads instead of GAT’s average operation. However, these methods all only rely on adjacent node pairs to calculate the attention weight, without considering that entity nodes may have different weights in different predicted links to contribute semantic information. In contrast, our method can give different attention weights to its neighbor nodes for the same drug entity according to the different prediction targets, so as to obtain its different semantic information. Similarly, the TAGNN (Ying *et al.*, 2021) uses a gated graph neural network to obtain node semantic information and uses a target-based attention layer based to calculate weights for full-graph nodes for recommendation. It does not combine the attention method with the topological structure of the graph, which makes it likely to overfit to discrete special nodes and thus not interpretable. Correspondingly, we construct an attention layer to generate attention pathways through the topology of each node to obtain node semantic information, which not only focus on different nodes according to different predicted links but also maintain interpretability.

*Knowledge graph-based models*. Knowledge graph (KG) is essentially described as a network with rich information, which can provide structural relationships among multiple entities, and unstructured semantic relationships related to each entity. Nowadays, many biomedical knowledge bases are published in the form of knowledge graphs (Whirl-Carrillo *et al.*, 2012), and some researchers have combined public datasets and biomedical corpora to build more complete biomedical knowledge graphs (Cong *et al.*, 2018; Harnoune *et al.*, 2021; Zheng *et al.*, 2021). The DDI prediction model developed based on these knowledge graphs has also achieved certain results. Karim *et al.* (2019) adopted the method of graph embedding for DDI prediction using KG. Lin *et al.* (2020) adopted an end-to-end and fully knowledge graph-based framework for DDI prediction. Yu *et al.* (2021) improves model performance and interpretability through a layer-independent graph attention mechanism. Chen *et al.* (2021) comprehensively consider the molecular features of drugs and the semantic information of knowledge graphs and fuse the drug representations of the two modalities in a novel way. Whether it is a graph embedding or a graph attention method, each drug can only obtain one embedding representation, which cannot reflect the semantic diversity of drugs in different drug pair links. Correspondingly, our method can generate different attention pathways according to different drug pairs, and generate different embedding representations for drug nodes, so as to obtain the potential different semantics of drugs.

## 3 Materials and methods

### 3.1 Overview

**Fig. 1.**
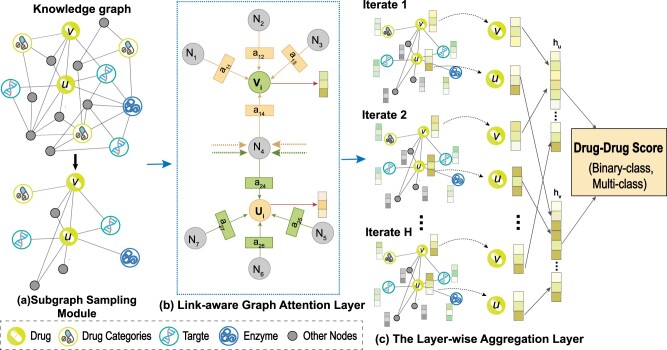
The Overview of LaGAT. (**a**) The Subgraph Sampling Module extracts the nodes in the KG according to the drug pair to form the subgraph corresponding to the drug. (**b**) After obtains the subgraph, LaGAT uses drug *v* as the query vector to calculate the attention weight (a11 to a14) of each node in the drug *u* subgraph, and uses drug *u* as the query vector to calculate the attention weight (a24 to a27) of each node in the drug *v* subgraph. Then, each node (*U_i_*, *V _i_*) of the two subgraphs aggregates the embedding representations of neighbor nodes according to the attention weights of its neighbor nodes, updating its own embedding representation. Then, LaGAT can get the updated embedding representation of all nodes including nodes *u*, *v*. (**c**) After iterating again *H* times, the layer-wise aggregation layer obtain multiple representations of drug *u*, *v* and concatenate the representations into a single vector to calculate the final DDI prediction score

As shown in Figure 1, our model can be decomposed into three parts: the subgraph sampling module, link-aware graph attention layer and the layer-wise aggregation layer. First, the subgraph sampling module extracts drug pairs from the dataset, forming a DDI matrix. At the same time, it obtains the *H*-hop neighbor nodes of each drug, and randomly selects *K* of each hop neighbor nodes to form a drug’s subgraph and embeds all the nodes contained in the drug subgraph. Then, the link-aware attention layer computes attention weights for the neighbor nodes of each node in the subgraph, making each node update its own embedding representation by aggregating the embedding representations of its neighbor nodes according to the attention weight. Finally, the layer-wise aggregation layer connects the embedding representation of each update of the drug node to obtain the final feature vector of the drug node and then predicts the label of the DDI.

### 3.2 Notation and problem formulation

Given a set of various biomedical entities *N_e_* and the biomedical relation *R* among the entities, the external biomedical KG is definedas $G=\left\{\left(h,r,t|h,t\in N_{e},r\in R\right)\right\}$. Each entity-relation-entity triples (h,r,t) describes a relation *r* between biomedical entity *h* and biomedical entity *t*. We present drug set as $D\subseteq N_{e}$ and define the DDI matrix $Y\in {\left(0,C\right)}^{\left(\left|D\right|*\left|D\right|\right)}$ with each element $y_{i,j}$ means the types of DDI relations, where *C* is the total number of types of DDI pairs. Note that we consider all types of DDI pairs and $y_{i,j}=0$ indicates the absence of evidence for interaction for the binary-class prediction task.

Our main task is to learn a prediction function ${\hat{y}}_{i,j}=\rho (i,j|\theta ,Y,G)$, where ${\hat{y}}_{i,j}$ denotes the probability that drug *j* will interact with drug *i*, and *θ* denotes the model parameters of function *ρ*.

### 3.3 The subgraph sampling module

Inspired by the GNN method (Hamilton *et al.*, 2017), we only select nodes with the *H*-hop distance from a drug to form a subgraph (the same definition as the GNN method), where *H* is a hyper-parameter. Usually the value of *H* will be greater than or equal to 1 to extract higher-order structures and semantic relations, so that we do not need to put the entire KG into training and still obtain the effective information from KG.

At the same time, in the real-world knowledge graph, the size of the neighbor node set $N_{\varepsilon }^{\text{neigh}}$ of each entity node *ε* may be very different. Inspired by the KGNN method (Lin *et al.*, 2020), we uniformly sample the sets $N_{\varepsilon }^{\text{neigh}}$ and get the set $N_{\varepsilon }(k)$ (here *k* is also a hyper-parameter, which determines the size of the receptive field of a single-layer entity). For any sample drug pair (*u*, *v*), sampling can get their 1-hop neighbors, sampling 1-hop neighbor nodes to get 2-hop neighbors from them, and so on. Then we can get a set of node $N_{u}(H,k),\;N_{v}(H,k)$ and get the desired subgraph *G_u_*, *G_v_* as input to the attention layer.

### 3.4 The link-aware graph attention layer

In this subsection, we describe the link-aware graph attention layer and compare it with the origin the GAT layer.

Generally, the GAT layer use the embedding representation of the node itself as the Query vector, combined with the embedding representation of its neighboring nodes to learn the attention weight. This strategy successfully makes each node assign different weights to each of its neighbors, but its limitation is that as long as the topology of a node is deterministic, the attention weights it eventually learns are deterministic. That is, GAT assumes that the attention pathway inside each drug subgraph is fixed, resulting in learned node features that can only capture a single semantic information. However, the link prediction task uses the label of the drug pair as a supervision signal. In different drug pairs, the nodes that each node needs to pay attention to are not fixed, that is, attention pathways in drug subgraphs should be variable based on drug pairs, and the semantic information of nodes may be diverse. In this case, the GAT layer may only learn the semantic information of nodes under most link labels or tend to evenly distribute attention weights, which may fail to learn a suitable attention weight for each neighbor node and hurt the interpretability of the model.

Algorithm 1 LaGAT algorithm

**Input**: Drug pair (*u*, *v*); subgraph $G_{u}(N_{e}^{u},R_{u}),G_{v}(N_{e}^{v},R_{v})$; neighborhood funtion $N:\varepsilon \to 2^{\varepsilon },\forall \varepsilon \in N_{e}^{u},N_{e}^{v}$; node embedding function *E*; depth *H*;

**Output**: Node vector representations *Z_u_*, *Z_v_*

1: **for**d∈{u,v}**do**

2:  **if** *d* is equal to *u* **then**

3:   q←E(v)

4:  **else**

5:   q←E(u)

6:  **end if**

7:  $z_{\varepsilon }^{0}\leftarrow E(\varepsilon ),\forall \varepsilon \in N_{e}^{d}$

8:  **for**h=1⋯H**do**

9:   **for**$\varepsilon \in N_{e}^{d}$**do**

10:    $\alpha_{j}\leftarrow q\cdot z_{j}^{h-1},\forall j\in N(\varepsilon )$

11:    $z_{N}\leftarrow \sum_{j\in N(\varepsilon )}\alpha_{j}\cdot z_{j}^{h-1}$

12:    $z_{\varepsilon }^{l}\leftarrow \mathrm{Aggregator}(z_{\varepsilon }^{h-1},z_{N})$

13:   **end for**

14:  **end for**

15:  $Z_{d}\leftarrow z_{d}^{h}$

16: **end for**

17: **return** *Z_u_*, *Z_v_*

So, we design link-aware graph attention layer to replace GAT layer. Algorithm 1 shows the pseudo-codes of applying LaGAT between given drug pairs. Specifically, for any sample drug pair(*u*, *v*), after obtain the subgraph *G_u_*, *G_v_*, link-aware graph attention layer produces a new set of node vector representations *Z_u_*, *Z_v_* as its output. When the node it computes belongs to *G_u_*, it uses the embedding representation of drug node *v* as the query vector (Lines 2–6). At the same time, it still uses the neighbor nodes of each node as the Key vector, and obtains the attention weight by calculating the inner product of the Query vector and the Key vector, which makes the neighbor nodes concerned by each node change according to the label. Among them, *α_j_* is the attention weight of the node embedding $z_{j}^{h-1}$, and query vector is the embedding representation of the drug node *ε* (Line 10). Then, it aggregates the embedding to obtain a vector *z_N_*, which is input to the Multilayer Perceptron (MLP) layer named aggregator to obtain the next embedding representation of node $z_{\varepsilon }^{h}$ (Line 11).

Aggregator is an important MLP layer in the link-aware graph attention layer. We implement similar multiple types of aggregators (Lin *et al.*, 2020) as follows: $\text{Concat}=\sigma (W\cdot \text{concat}(z_{\varepsilon }^{h-1},z_{N})+b)$ and $\text{Neighbor}=\sigma (W\cdot z_{N}+b)$. We use LeakyReLU (α=0.1) as the activation function in the above method. After evaluating the results of the three methods, we finally chose the neighbor form of aggregation. We will show the evaluation results of the two methods in more experimental results (Section 4.5).

### 3.5 The layer-wise aggregation layer

After iterating again *H* times, we obtain multiple representations *Z_u_* for drug node *u*, namely $\left\{z_{u}(1),\ldots ,z_{u}(H)\right\}$; analogous to another drug node *v*, $\left\{z_{v}(1),\ldots ,z_{v}(H)\right\}$ are obtained. As we mentioned earlier and shown in Figure 1, $z_{u}(i)$ represents different depths of high-level structure and semantic information. We hence adopt the layer-aggregation layer (Xu *et al.*, 2018), to concatenate the representations at each step into a single vector, as follows:

(1) $$z_{u}=[z_{u}^{\left(0\right)}||z_{u}^{\left(1\right)}||z_{u}^{\left(2\right)}\ldots ||z_{u}^{\left(H\right)}]$$

(2) $$z_{v}=[z_{v}^{\left(0\right)}||z_{v}^{\left(1\right)}||z_{v}^{\left(2\right)}\ldots ||z_{v}^{\left(H\right)}]$$

In this way, we try our best to aggregate high-level structure information together, which contains more information than just relying on the embedding representation of the last iteration.

Finally, we calculate the inner product of the two drug representations and obtain the final DDI prediction score through the activation function:

(3) $${\text{score}}_{b}=\sigma (z_{u}^{T}z_{v})$$

(4) $${\text{score}}_{m}=\delta (\mathrm{MLP}(z_{u}||z_{v}))$$

For binary classification tasks, we use score_*b*_, where *σ* is the sigmoid activation function; for multi-classification tasks, we use score_*m*_, where *δ* denotes softmax. Both scores represent the possibility of drug pair interaction.

### 3.6 Training

Through the representation learned in the previous part, we can integrate all the information from the drug and the topological neighborhood to predict the interaction value between the drug–drug pair. We use Xavier (Glorot and Bengio, 2010) to initialize the training parameters in all layers (including the embedding layer).

For a given set of drug–drug pairs and the true interaction value in the training dataset, we use binary cross-entropy as the loss function cross, the formula is as follows:

(5) $$\text{Loss}=\sum_{(i,j)\in Y}-y_{i,j}\;\text{log}\;{\hat{y}}_{i,j}-(1-y_{i,j})\;\text{log}(1-{\hat{y}}_{i,j})$$

where ${\hat{y}}_{i,j}$ is the predicted value, $y_{i,j}\in (0,1)$ is the ground-truth value, and *Y* represents the set of drug–drug pairs. During training, we learn the model parameter by minimizing the loss using stochastic gradient optimizers such as Adam (Glorot and Bengio, 2010)

## 4 Experiment

### 4.1 Datasets and settings

*Datasets*. We use two real-world datasets to evaluate the KGSAT we proposed. (i) **Binary-class DDIs**: We use the data from the KEGG-drug provided by (Lin *et al.*, 2020) to construct KG including DDI matrix, which contains 1925 approved drugs and 56 983 approved interactions. We randomly generated negative samples at a ratio of 1:1, where negative samples refer to drug pairs that have not appeared in the positive samples. (ii) **Multi-class DDIs**: We use the data provided by (Lin *et al.*, 2020) from the Drugbank to construct KG and the data provided by (Yu *et al.*, 2021), which contains 1709 drugs mapped to DrugBank identifiers (IDs) and 136351 drug pairs involving 86 types of pharmacological relationships between drugs.

*Experimental settings*. For the binary-class, experimental results are presented in following metrics: the accuracy (ACC), the area under the receiver operating characteristic (AUC), the area under the Precision–Recall curve (AUPR), the F1 score. And we randomly divide the dataset KEGG into 5 folds, 4 of which are training sets, and the remaining one is equally divided into validation set and test set, so that 5-fold cross-validation is performed. For the multi-classification tasks, we use ACC and Macro-F1 as performance metrics. Macro-F1 is the average of the F1 scores for each category, and the samples of the Drugbank dataset are the same as Yu *et al.* (2021).

We use Adam algorithm for parameter training, and perform a grid search on our model on two important hyperparameters, node dimension and number of neighbor samples. We finally use a node embedding dimension of 64 and a neighborhood sampling number of 64 as parameters on the KEGG dataset. On the Drugbank dataset, we finally use a combination of a node embedding dimension of 32 and a neighborhood sampling number of 128. The number of training epochs is set to 50, and other hyper-parameter settings are shown in the Table 1, which are optimized on the validation set by AUCROC on the binary classification task and by ACC on the validation set on the multi-classification task.

**Table 1.** Hyper-parameter settings

Parameters	Setting
Batch size	1024
Learning rate	1e–2
L2 weight	1e–7
H-hop	1

### 4.2 Baselines

We constructed several strong baseline models according to the original paper: KGNN, GAT and GAT-const, and performed grid search on each model on two important hyperparameters, node dimension and neighbor sampling number, and found keeping the rest of the parameters consistent with our method allows a relatively fair discussion of the effectiveness of our proposed attention method.

1. KGNN: (Lin *et al.*, 2020) through GNN and external KG, the neighborhood information of each node is sampled and aggregated from the local receiver of each node for DDI prediction. On KEGG, we end up using a node embedding dimension of 16 and a neighborhood sampling number of 64 as parameters for GAT and GAT-const. On Drugbank, we end up using a node embedding dimension of 32 and neighborhood sampling number 32 as the GAT and GAT-const parameters. Parameters of GAT-const.
2. GAT: (Veličković *et al.*, 2018) A multi-head attention mechanism is adopted to assign multiple attention weights to each neighbor node through multiple attention heads. At the same time, they also proposed a model GAT-const that assigns the same weight to each node to compare the effectiveness of GAT. On KEGG, we finally set the number of attention heads for GAT to 8, and use the node embedding dimension to be 64 and the number of neighborhood samples to be 64 as the parameters of GAT and GAT-const. On Drugbank, we finally set the attention of GAT to The number of heads is 2, and the node embedding dimension is 32 and the number of neighborhood samples is 128 as parameters for GAT and GAT-const.
3. SumGNN: (Yu *et al.*, 2021) propose a GNN based method, which is enabled by a subgraph extraction module, a self-attention based subgraph summarization scheme to generate reasoning pathway within the subgraph, and a multichannel knowledge and data integration module. Unlike other models, we directly use the results of the original SumGNN paper for discussion.

### 4.3 Results and analysis

We compare the performance of the proposed method with the baseline we mentioned. Table 2 reports AUC, ACC, F1 scores, AUPR on the KEGG-drug dataset and ACC, F1 scores on the Drugbank dataset. Compared to all baselines, our model performs the best. More specifically, on KEGG-drug, GAT and GAT-const are not significantly different while outperform KGNN, and our model improves by 2.54% on AUC-ROC, 3.02% on ACC, 3.09% on F1 and 1.98% on AUPR compared to the GAT. On Drugbank, GAT and GAT-const are equally competitive with SumGNN, and our model improves by 4.3% on ACC and 4.14% on F1 compared to the GAT. These findings demonstrate the effectiveness of our modele.

**Table 2.** The performance of baselines, LaGAT-base and LaGAT on two datasets, where LaGAT-base removes layer aggregation layers on the basis of LaGAT

Methods	Binary_class: KEGG-drug				Multi_class: DrugBank	
	AUC	ACC	F1	AUPR	ACC	Marco-F1
KGNN	95.63 ± 0.18	90.08 ± 0.27	90.32 ± 0.22	94.20 ± 0.32	93.03 ± 0.07	84.56 ± 1.36
GAT	97.42 ± 0.14	92.88 ± 0.21	92.97 ± 0.18	96.67 ± 0.30	91.74 ± 0.15	88.75 ± 1.89
GAT-const	97.68 ± 0.22	93.22 ± 0.51	93.37 ± 0.56	97.10 ± 0.27	91.71 ± 0.04	87.66 ± 0.86
SumGNN^a^	–	–	–	–	92.66 ± 0.14	86.85 ± 0.44
LaGAT-base	98.33 ± 0.08	94.94 ± 0.10	95.04 ± 0.11	97.70 ± 0.19	95.71 ± 0.23	89.25 ± 1.03
**LaGAT**	**98.96 ± 0.10**	**95.90 ± 0.21**	**95.96 ± 0.20**	**98.95 ± 0.21**	**96.04 ± 0.08**	**92.89 ± 0.91**

*Note*: We used a 5-fold cross-validation with random split, and reported the average and standard deviation. Bold numbers signify the best performance for each metric column.

a The experimental results of SumGNN are from Yu *et al.* (2021).

**Fig. 2.**
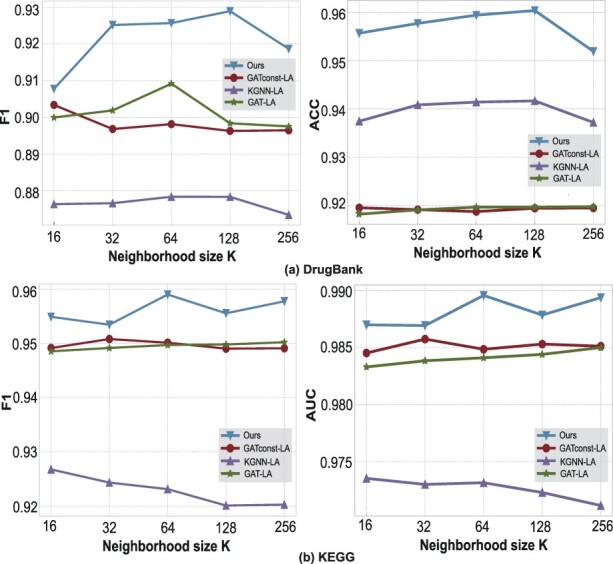
The performance of all models using layer aggregation layers. LaGAT still performs best. (**a**) Midazolam and Cyclosporine. (**b**) Midazolam and Amobarbital

In order to further explore the effectiveness of our designed attention method, we remove layer aggregation layers to get LaGAT-base. As shown in Table 2, LaGAT-base still significantly outperforms all baseline models. Meanwhile, we add layer aggregation layer to GAT, GAT-const and KGNN, respectively, and then conduct experiments on two datasets with different neighborhood sampling numbers *k*. The result is shown in Figure 2.

Combining the results in Table 2 and Figure 2, it can be seen that the performance of all models has been improved after adding the layer aggregation layer, and LaGAT is still significantly better than other models on both datasets, which shows that our proposed attention method is effective and does not depend on the layer aggregation layer. Among them, the performance of GAT and GAT-const still did not show a statistically significant difference under each parameter. This supports our hypothesis that when nodes need to notice different neighbors based on labels, GAT may degenerate into assigning the same weight to each node. At the same time, the way KGNN uses the inner product of the embedded representation of nodes and edges as the attention score is not better than GAT-const, which is why we do not use a similar way to introduce edge features.

### 4.4 Case study

**Fig. 3.**
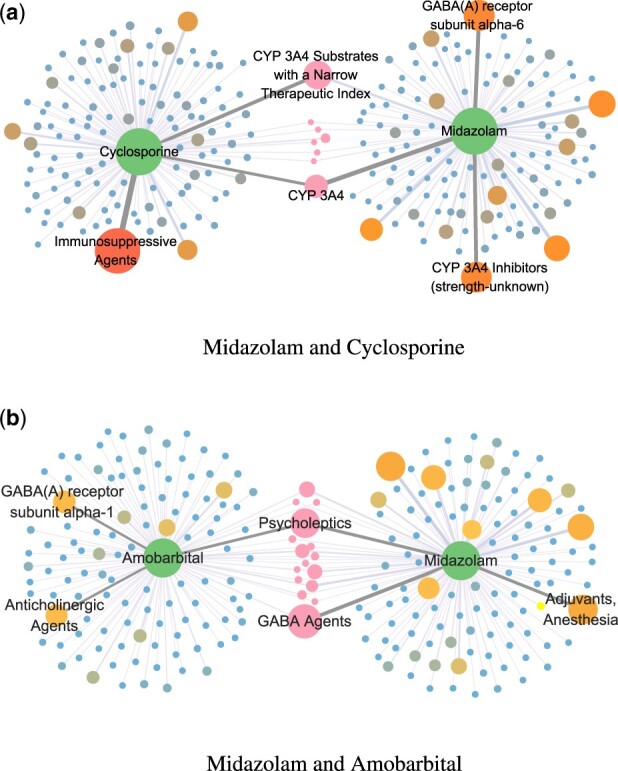
LaGAT generates attention pathways to show why models predict DDI types. For each drug node, we annotate the top-3 neighbor nodes with attention weights and bold the connected edges. For the common neighbor nodes in the two drugs, we take the maximum value of its weight, and then use the node size to indicate the attention weight of each node

We visualize the predict results of the model on the drugbank test set, and choose a common drug, Midazolam, as an example to show how our proposed attention method improves the accuracy of model predictions. Midazolam is a short-acting injectable benzodiazepine with rapid onset that is commonly used in seizures, anesthesia and anxiety disorders. We visualized the attention pathways generated by the model for two different types of DDI pairs picked from a drug pair collection containing Midazolam, as Figure 3 shown. We see that the model only assigns high attention weights to a small number of nodes, so we only label the top three nodes for each drug attention weight.

For Midazolam and Cyclosporine, LaGAT assigns highest probability for the DDI type ’The serum concentration of drug one can be increased when it is combined with drug two’. Among the nodes common to both drugs, the model assigns the highest attention weight to the enzyme node, CYP 3A4. Studies have shown that cyclosporine is metabolized in the gut and liver by CYP450 enzymes, mainly contributed by CYP 3A4 and CYP 3A5, which competitively inhibits CYP3A4 in human liver microsomes (Amundsen *et al.*, 2012; Saiz-Rodríguez *et al.*, 2020). Meanwhile, midazolam biotransformation is mediated by CYP 3A4 with highly variable activity (Denisov *et al.*, 2021; Gascon, 1991), which supports the predictions of our model.

For Midazolam and Amobarbital, LaGAT assigns highest probability for the DDI type ’The risk or severity of adverse effects can be increased when Midazolam is combined with Amobarbital’. We note that among nodes common to both drugs, the model assigns the highest attention weight to the drug category node, Psycholeptics. In addition, the high attention nodes that the model pays attention to are the target node of Amobarbital, GABA(A) receptor subunit alpha-1 and the drug categorie node of Midazolam, GABA Agents. Studies have shown that amobarbital (like all barbiturates) works by binding to GABAA receptors at the alpha or beta subunit (Kim and Mathers, 2004; Zhu *et al.*, 2018). Like benzodiazepines, barbiturates potentiate the action of GABA on this receptor, but at a site that is different from the binding site for GABA itself and from the benzodiazepine binding site, which supports the predictions of our model.

Noting that the model correctly predicts the DDI types of Midazolam and Amobarbital both before and after using our attention method. But for Midazolam and Cyclosporine, the model predicts correctly only after using our method. Meanwhile, the top-3 nodes with attention weights computed by LaGAT for Midazolam are completely different in the two DDI predictions. This supports our hypothesis that generating different attention pathways for the same drug according to different prediction targets can capture its different semantic information and help the model make correct DDI inferences.

### 4.5 Ablation experiment

**Fig. 4.**
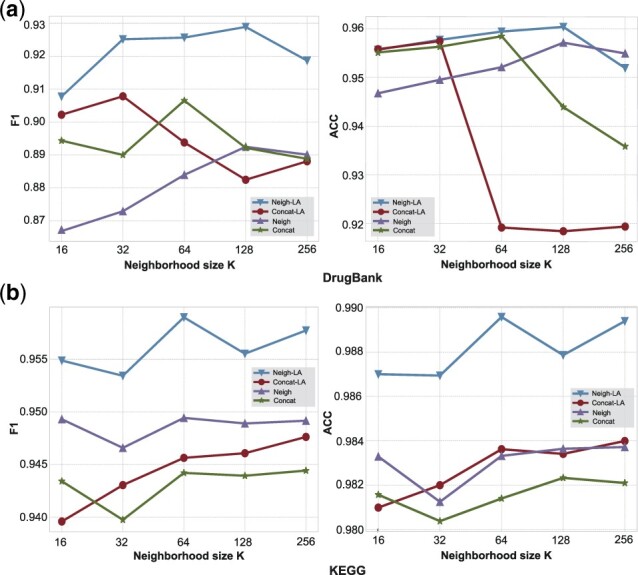
The performance of different variants of the model under different neighbor sampling size *K*

To verify the effect of layer aggregation layer and aggregation method on performance, we conduct a series of ablation studies on our proposed model on two datasets. As mentioned above, we implemented two types of aggregation as aggregation methods, corresponding to Neigh-LA and Concat-LA in the following figures, respectively. We then remove the layer aggregation layer for these two models to obtain Neigh and Concat, and the final result is shown in Figure 4.

The results show that the neigh method performs better than the concat method on KEGG and the opposite on Drugbank. However, the layer aggregation layer can significantly improve the model using the neigh method, which may be because the concat method and the layer aggregation layer reuse the initial embedding representation of the node under the Hop parameter of 1. Note that although using the concat method without the layer aggregation layer can have better results on Drugbank, we still choose the neigh method as the aggregation method for LaGAT-base and show the results in Section 4.3, because the other models compared in Section 4.3 all use the neigh method as the aggregation layer.

### 4.6 Parameter studies

**Fig. 5.**
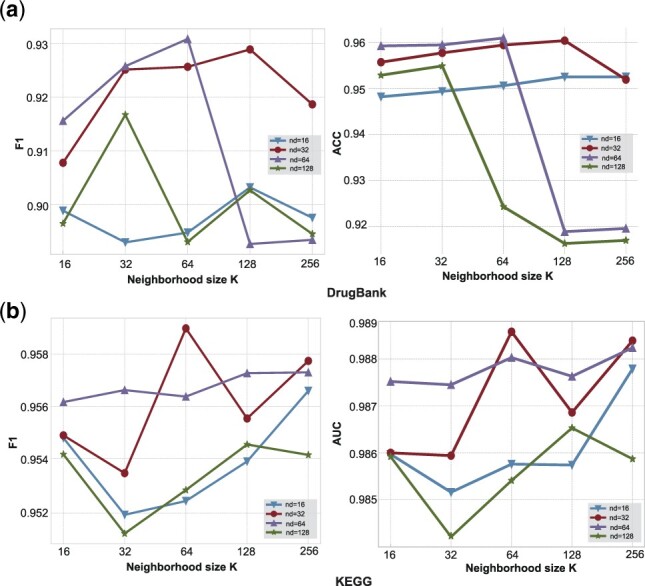
Model performance under different neighbor sample sizes *K* and node embedding dimension settings

In this section, we investigate the effect of node embedding dimension and the number of neighborhood samples on our model. On KEGG, we set the model’s node embedding dimensions nd = 32, 64, 128, 256, 512, and explore the performance of the model in the neighborhood sampling number *k* = 8, 16, 32, 64, 128, 256 under each dimension; on Drugbank, we set the model separately The node embedding dimensions of nd = 16, 32, 64, 128, 256, explore the performance of the model in the neighborhood sampling number *k* = 8, 16, 32, 64, 128, 256 under each dimension; the remaining parameters are the same as those in Section 4.1. Figures 5 report the various metrics of our model on both datasets.

*Influence of neighborhood sampling size*. We observe that appropriately increasing *K* can improve the performance of the model when the number of neighborhood samples *k* is small. This may be caused by insufficient capacity to contain semantic information when *k* is too small. With the further increase of *k*, the performance of the model on KEGG stabilizes, and the performance of the model on drugbank decreases greatly. We believe the reason is that the number of neighbors for each drug node in the drugbank dataset is about 100–300, while in the kegg dataset it is 10–100. When *K* exceeds the number of neighbors, the model will resample, so the model performance tends to be stable on kegg. However, on drugbank, considering too many different neighbors at the same time may make the model more likely to overfit some special neighbor nodes, which leads to a sharp drop in model performance when *K* is too large.

*Influence of dimension of node embedding*. The results show that an appropriate node dimension can make the model achieve the best performance. A too large node dimension will lead to overfitting of the model, and when *k* is large, the model performance is prone to a large decline. Too small node dimension is not enough to express enough semantic information. For the consideration of parameters and model stability, we finally chose 32 as the node embedding dimension on the drugbank data.

## 5 Conclusion

In this article, we propose a link-aware graph attention network for binary and multi-class drug interaction prediction tasks. It is able to generate different attention pathways for the same drug entity in different DDIs, providing interpretability for the results predicted by the model on KG. Experimental results on two real-world datasets demonstrate the effectiveness of our proposed method compared to several state-of-art works using KG. In addition, the visualization results of case study shows that our attention method can learn more accurate semantics, which is beneficial for the model to predict different types of DDIs. In our work, we mainly focus on extracting features from KGs via a plug-and-play link-aware graph attention layer. However, our method still suffers from the cold start problem. In the future, we will try to use the molecular features of drugs to perform feature fusion to solve this problem and further improve the model performance.

## Supplementary Material

btac682_Supplementary_Data

## References

[btac682-B1] Amundsen R. et al (2012) Cyclosporine a- and tacrolimus-mediated inhibition of cyp3a4 and cyp3a5 in vitro. Drug Metab. Dispos., 40, 655–661. 22205779 10.1124/dmd.111.043018

[btac682-B2] Asada, M. et al (2018). Enhancing drug-drug interaction extractionfrom texts by molecular structure information. In Proceedings of the 56th Annual Meeting of the Association for Computational Linguistics(Volume 2: Short Papers), pages 680–685, Melbourne, Australia.Association for Computational Linguistics.

[btac682-B3] Bahdanau D. et al (2014) Neural machine translation by jointly learning to align and translate. arXiv, 0473

[btac682-B4] Chen Y. et al (2021) Muffin: multi-scale feature fusion for drug–drug interaction prediction. Bioinformatics, 37, 2651–2658. 10.1093/bioinformatics/btab169 33720331

[btac682-B5] Cong Q. et al (2018) Constructing biomedical knowledge graph based on semmeddb and linked open data. In: *2018 IEEE International Conference on Bioinformatics and Biomedicine (BIBM), Madrid, Spain*. IEEE, pp. 1628–1631.

[btac682-B6] Denisov I. et al (2021) Midazolam as a probe for drug–drug interactions mediated by cyp3a4: homotropic allosteric mechanism of site-specific hydroxylation. Biochemistry, 60, 1670–1681. 34015213 10.1021/acs.biochem.1c00161 PMC8857940

[btac682-B7] Gao Z. et al (2022) Kg-predict: a knowledge graph computational framework for drug repurposing. J. Biomed. Inf., 132, 104133. 10.1016/j.jbi.2022.104133 PMC9595135 35840060

[btac682-B8] Gascon M.P. (1991) In vitro forecasting of drugs which may interfere with the biotransformation of midazolam. *Eur J Clin Pharmacol.*, 41(6) 573–8. 10.1007/BF00314987 1815969

[btac682-B9] Glorot X. , Bengio Y. (2010) Understanding the difficulty of training deep feedforward neural networks. In: Teh,Y.W. and Titterington,M. (eds.) *Proceedings of the Thirteenth International Conference on Artificial Intelligence and Statistics*, volume 9 of *Proceedings of Machine Learning Research*, Chia Laguna Resort, Sardinia, Italy. PMLR, pp. 249–256.

[btac682-B10] Hamilton W.L. et al (2017) Inductive representation learning on large graphs. In: Proceedings of the 31st International Conference on Neural Information Processing Systems, NIPS’17. Curran Associates Inc., Red Hook, NY, USA, pp. 1025–1035

[btac682-B11] Harnoune A. et al (2021) Bert based clinical knowledge extraction for biomedical knowledge graph construction and analysis. Comput. Methods Programs Biomed. Update, 1, 100042.

[btac682-B12] Huang K. et al (2020) Caster: predicting drug interactions with chemical substructure representation. AAAI, 34, 702–709.

[btac682-B13] Jin B. et al (2017) Multitask dyadic prediction and its application in prediction of adverse drug–drug interaction. AAAI, 31,1367–373.

[btac682-B14] Karim M.R. et al (2019) Drug–drug interaction prediction based on knowledge graph embeddings and convolutional-LSTM network. In: *Proceedings of the 10th ACM International Conference on Bioinformatics, Computational Biology and Health Informatics*, BCB ’19. Association for Computing Machinery, New York, NY, USA, pp. 113–123.

[btac682-B15] Kim H.S. , Mathers D.P.E. (2004) Selective GABA-receptor actions of amobarbital on thalamic neurons. *Br J Pharmacol*., 2004 Oct, **143**(4), 485–94. 10.1038/sj.bjp.0705974 PMC1575418 15381635

[btac682-B16] Lin X. et al (2020) KGNN: knowledge graph neural network for drug–drug interaction prediction. In: Bessiere C. (ed.) *Proceedings of the Twenty-Ninth International Joint Conference on Artificial Intelligence, IJCAI-20*. International Joint Conferences on Artificial Intelligence Organization. Main track, pp. 2739–2745.

[btac682-B17] Ma T. et al (2018) Drug similarity integration through attentive multi-view graph auto-encoders. In: *Proceedings of the 27th International Joint Conference on Artificial Intelligence, Stockholm, Sweden*, pp. 3477–3483.

[btac682-B18] Nyamabo A.K. et al (2021) Drug–drug interaction prediction with learnable size-adaptive molecular substructures. Brief. Bioinformatics, 22, bbab441. 10.1093/bib/bbab441 34695842

[btac682-B19] Ryu J.Y. et al (2018) Deep learning improves prediction of drug–drug and drug–food interactions. Proc. Natl. Acad. Sci. USA, 115, E4304–E4311. 29666228 10.1073/pnas.1803294115 PMC5939113

[btac682-B20] Saiz-Rodríguez M. et al (2020) Effect of the most relevant cyp3a4 and cyp3a5 polymorphisms on the pharmacokinetic parameters of 10 cyp3a substrates. Biomedicines, 8, 94. 32331352 10.3390/biomedicines8040094 PMC7235792

[btac682-B21] Takeda J. et al (2017) Predicting drug–drug interactions through drug structural similarities and interaction networks incorporating pharmacokinetics and pharmacodynamics knowledge. *J. Cheminform., *9, 16. https://doi.org/10.1186/s13321-017-0200-8. 10.1186/s13321-017-0200-8 PMC5340788 28316654

[btac682-B22] Vashishth S. et al (2019) Attention interpretability across NLP tasks. arXiv, 11218

[btac682-B23] Veličković P. et al (2018) Graph attention networks. In: *International Conference on Learning Representations, vancouver, Canada*.

[btac682-B24] Vilar S. et al (2014) Similarity-based modeling in large-scale prediction of drug–drug interactions. Nat Protoc., 2014 Sep, 9(9), 2147–63. 25122524 10.1038/nprot.2014.151 PMC4422192

[btac682-B25] Whirl-Carrillo M. et al (2012) Pharmacogenomics knowledge for personalized medicine. Clin. Pharmacol. Ther., 92, 414–417. 22992668 10.1038/clpt.2012.96 PMC3660037

[btac682-B26] Wiegreffe S. , Pinter Y. (2019) Attention is not explanation. arXiv, 10186

[btac682-B27] Xu K. et al (2018) Representation learning on graphs with jumping knowledge networks. arXiv, 03536

[btac682-B28] Ying C. et al (2021) Do transformers really perform bad for graph representation? arXiv, 05234

[btac682-B29] Yu Y. et al (2021) SumGNN: multi-typed drug interaction prediction via efficient knowledge graph summarization. Bioinformatics, 37, 2988–2995. 10.1093/bioinformatics/btab207 PMC10060701 33769494

[btac682-B30] Zhang J. et al (2018) GaAN: gated attention networks for learning on large and spatiotemporal graphs. arXiv,07294

[btac682-B31] Zheng S. et al (2021) PharmKG: a dedicated knowledge graph benchmark for biomedical data mining. Brief. Bioinformatics, 22, bbaa344. 33341877 10.1093/bib/bbaa344

[btac682-B32] Zhu S. et al (2018) Structure of a human synaptic GABAA receptor. Nature, 559, 67–72. 29950725 10.1038/s41586-018-0255-3 PMC6220708

[btac682-B33] Zitnik M. et al (2018) Modeling polypharmacy side effects with graph convolutional networks. Bioinformatics, 34, i457–i466. 29949996 10.1093/bioinformatics/bty294 PMC6022705

