# Supplementary material for: LaGAT: link-aware graph attention network for drug–drug interaction prediction
Source: Bioinformatics. 2022 Oct 22;38(24):5406–12. doi: 10.1093/bioinformatics/btac682 (PMC9750103; doi:10.1093/bioinformatics/btac682)
Supplement: btac682_Supplementary_Data [file btac682_supplementary_data.pdf]

1 Model Architecture

Our model can be decomposed into three parts: the subgraph sampling module, link-aware graph attention layer, and the layer-wise aggregation layer. The only trainable neural network layer is the link-aware graph attention layer (LaGAT layer). And for each layer of link-aware graph attention layer built, the sampling depth of the model Hop will also increase by 1. Table 1 shows the performance of models built with one-layer LaGAT($Hop = 1$) and models built with two-layer LaGAT($Hop = 2$) on the KEGG-drug dataset.

Dataset	Size of Hop H	
	1	2
KEGG-drug	95.90	95.17
DrugBank	95.74	95.46

Table 1. The effect of the number of LaGAT layers on the ACC of the model.

We noticed that the performance of the model at Hop 1 has reached the optimum, so in the end we chose to build a model architecture with only one layer of LaGAT.

2 Training Time

Table 2 shows the training time of the model and the sampling time of subgraphs under different sampling depth Hop. As mentioned earlier, the model performance does not improve significantly when Hop is 2. And due to code design, when the sampling frequency of neighbors is K, the GPU memory usage is proportional to K^H. So we only tested the model when Hop=1 and Hop=2.

Dataset	Size of Hop H	
	1	2
KEGG-drug	42 s	99 s
DrugBank	61 s	166 s

Table 2. The running time of LaGAT for two datasets.

3 Dataset Details

3.1 Drug Frequency Distribution

Figure 1 and Figure 2 are the Drug frequency distribution of the two datasets we used, Drugbank and KEGG-drug, respectively. The abscissa represents the drug-related DDI sample number interval, and the ordinate represents the number of drugs included in the interval. We can see that the number of samples associated with different drugs varies greatly, whether it is KEGG-drug or Drugbank. A few drug nodes have 500-1000 DDI samples, most drug nodes only have 50-100 DDI samples, and even many drugs have less than 10 DDI samples.

For the KEGG-drug dataset, our positive samples are derived from the recorded DDI relations in the KEGG database, and the negative samples are randomly selected by us according to the unrecorded DDI relations in KEGG. In particular, Figure 3 shows the positive samples of KEGG-drug. Compared with Figure 2, it can be seen that the drug frequency distribution of KEGG-drug has changed after the negative samples are generated. This is because, for the KEGG-drug dataset, the number of negative samples related to each drug is randomly selected, as shown in Figure 4.

We tried a new negative sample generation scheme, which maintains the drug frequency distribution of KEGG-drug positive samples to generate negative samples, and thus generates a new binary dataset KEGG-m. We conducted experiments under the same parameters using our model LaGAT, and the experimental results are reported in Table 3 (KEGG-m). It can be seen that although the AUC and AUPR of the model are only slightly reduced, the ACC and F1 scores drop significantly. The reason is that since the number of positive samples associated with some drugs is small, after generating negative samples with the same number, the total number of samples associated with drugs is very small. For these drugs, the model cannot learn good features to make accurate DDI predictions.

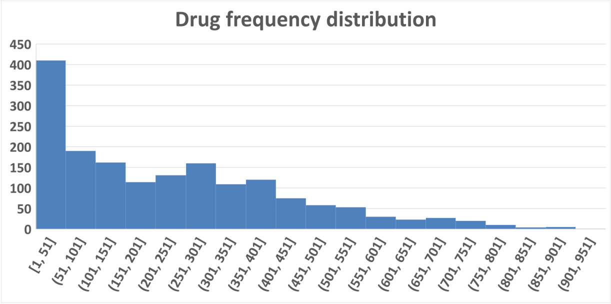

Fig. 1: Drugbank

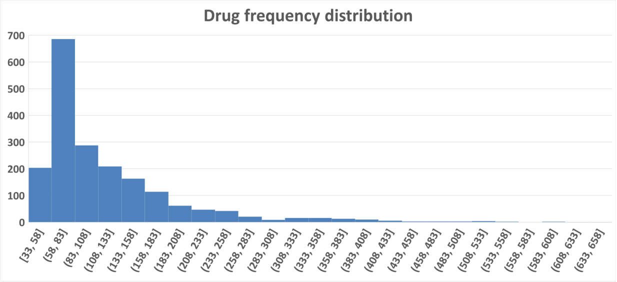

Fig. 2: KEGG-drug

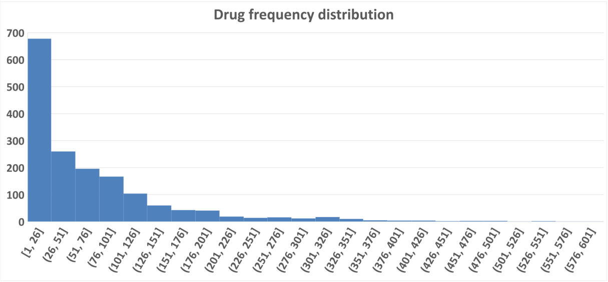

Fig. 3: Positive samples of KEGG-drug

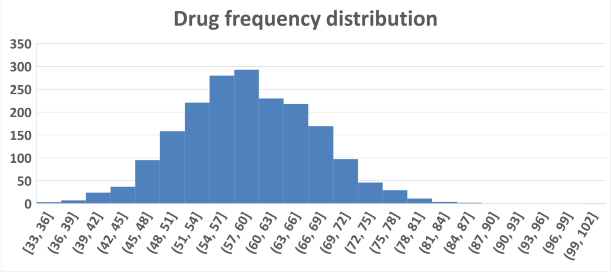

Fig. 4: Negative samples of KEGG-drug

Dataset	Metric			
	AUC	ACC	F1	AUPR
KEGG	98.96 ± 0.30	95.90 ± 0.21	95.96 ± 0.20	98.65 ± 0.21
KEGG-m	97.88 ± 0.14	92.96 ± 0.27	92.98 ± 0.25	98.00 ± 0.16
KEGG-u	98.59 ± 0.08	95.13 ± 0.20	95.20 ± 0.23	98.24 ± 0.16

Table 3. The performance of LaGAT on a dataset of binary classification tasks with 5-fold cross-validation. Where KEGG represents the experiment on the dataset (original dataset) consisting of randomly generated negative samples, KEGG-rc represents the experiment on the dataset consisting of negative samples generated according to the drug frequency distribution of KEGG positive samples, KEGG-pr represents the experiment with 5-fold cross-validation without random splits on the original dataset. We used a 5-fold cross validation with random split, and reported the average and standard deviation.

3.2 Methods of Splitting Datasets

For the binary classification task, we randomly divided the dataset into 5 folds, one of which was randomly selected and randomly divided into test set and validation set by 1:1, so as to perform 5-fold cross-validation. We implemented two partitioning methods, the first is to keep the dataset randomly divided into 5 folds (used in the main text), so that the drug distribution in each fold is consistent. The second is to divide the drug into 5 folds, where each fold contains drugs that the other folds do not have, so that we can test the model's ability to generalize to drugs that have not been seen before. In the second approach, we specify that 20 percent of the drugs contained in each fold are unique and not present in other folds. Moreover, we counted the number of samples related to these unique drugs, and the average number of samples related to their own unique drugs for each fold is about 20 percent. The experimental results are recorded in Table 3 (KEGG-u), we can see that compared with the first one (KEGG in Table 3), the performance of the model has declined, but it still maintains a high level. It can be seen that the model still maintains good generalization performance when making DDI predictions for a small number of unseen drugs.
